# Synergistic Bactericidal Effects of Quaternary Ammonium Compounds with Essential Oil Constituents

**DOI:** 10.3390/foods13121831

**Published:** 2024-06-11

**Authors:** Adrián Pedreira, Susana Fernandes, Manuel Simões, Míriam R. García, José Antonio Vázquez

**Affiliations:** 1 Group of Recycling and Valorization of Waste Materials (REVAL), Spanish National Research Council (IIM-CSIC), Rúa Eduardo Cabello 6, 36208 Vigo, Spain; jvazquez@iim.csic.es; 2 Biosystems and Bioprocess Engineering Group (Bio2Eng), Spanish National Research Council (IIM-CSIC), Rúa Eduardo Cabello 6, 36208 Vigo, Spain; miriamr@iim.csic.es; 3 LEPABE—Laboratory for Process Engineering, Environment, Biotechnology and Energy, Faculty of Engineering, University of Porto, Rua Dr. Roberto Frias, 4200-465 Porto, Portugal; sfernandes@fe.up.pt (S.F.); mvs@fe.up.pt (M.S.); 4 ALiCE—Associate Laboratory in Chemical Engineering, Faculty of Engineering, University of Porto, Rua Dr. Roberto Frias, 4200-465 Porto, Portugal

**Keywords:** antimicrobial synergism, benzalkonium chloride, didecyldimethylammonium chloride, carvacrol, eugenol

## Abstract

Antimicrobial tolerance is a significant concern in the food industry, as it poses risks to food safety and public health. To overcome this challenge, synergistic combinations of antimicrobials have emerged as a potential solution. In this study, the combinations of two essential oil constituents (EOCs), namely carvacrol (CAR) and eugenol (EUG), with the quaternary ammonium compounds (QACs) benzalkonium chloride (BAC) and didecyldimethylammonium chloride (DDAC) were evaluated for their antimicrobial effects against *Escherichia coli* and *Bacillus cereus*, two common foodborne bacteria. The checkerboard assay was employed to determine the fractional inhibitory concentration index (FICI) and the fractional bactericidal concentration index (FBCI), indicating the presence of bactericidal, but not bacteriostatic, synergy in all QAC–EOC combinations. Bactericidal synergism was clearly supported by Bliss independence analysis. The bactericidal activity of the promising synergistic combinations was further validated by time–kill curves, achieving a >4-log_10_ reduction of initial bacterial load, which is significant compared to typical industry standards. The combinations containing DDAC showed the highest efficiency, resulting in the eradication of bacterial population in less than 2–4 h. These findings emphasize the importance of considering both bacteriostatic and bactericidal effects when evaluating antimicrobial combinations and the potential of EOC–QAC combinations for sanitization and disinfection in the food industry.

## 1. Introduction

Antimicrobial compounds are commonly used in the food industry to control the growth and spread of harmful and spoilage microorganisms, serving either as disinfectants and sanitizers for equipment and facilities or as preservatives when in direct contact with food [1,2]. However, some microorganisms have demonstrated the ability to develop tolerance mechanisms that allow them to survive exposure to antimicrobial compounds.

Antimicrobial tolerance in the food industry is a major concern due to its potential to cause persistent contamination and increase the risk of foodborne illnesses. Tolerant bacteria can survive antimicrobial treatments that would typically inhibit or kill them, making it harder to maintain sanitary conditions [3,4]. Additionally, these bacteria can serve as reservoirs for resistance genes, contributing to the emergence and spread of antimicrobial-resistant strains, which are more difficult to control and eliminate, posing a significant threat to public health and food safety [5]. This phenomenon not only undermines the efficacy of disinfection procedures but also raises concerns about the potential for cross-resistance to antibiotics, further exacerbating the public health implications [1]. Consequently, new and innovative strategies are needed to combat it.

One potential approach to deal with tolerant strains is the use of synergistic combinations, where two or more antimicrobial agents are combined to enhance their efficacy and overcome tolerance mechanisms [6].

Carvacrol (CAR) and eugenol (EUG) are essential oil constituents (EOCs) commonly employed as flavoring additives in foods [7,8,9]. Their use is also authorized in the USA and the EU in the formulation of cleaning products, disinfectants and pest control biocides, air care products, and cosmetics, among others [10,11,12,13]. In spite of their proven antimicrobial effect, most EOCs are water-insoluble, which limits their employment as active compounds in the formulation of disinfectants.

Didecyldimethylammonium chloride (DDAC) and benzalkonium chloride (BAC) are quaternary ammonium compounds (QACs) widely used as disinfectants [14,15]. USA and UE legislation authorized the inclusion of DDAC and BAC in the formulation of biocides intended for the food industry, household products, and veterinary area [16,17,18,19]. QACs are cationic detergents and can serve as effective single-stage cleaning and disinfection agents in situations with light soiling [20]. Their surfactant properties enable them to lower surface tension, facilitating the formation of micelles and increasing their dispersibility.

A notable concern related to the use of QAC is the emergence of bacterial clones showing reduced susceptibility as a result of exposure to sub-inhibitory concentrations and, even more alarmingly, the acquisition of cross-resistance to other biocides and antibiotics [21]. Interestingly, phytochemicals such as EOCs are resistance-modifying agents [22] and could potentially avoid the emergence of resistance in QACs-exposed bacteria.

Hence, the antimicrobial activity of QAC–EOC combinations is very interesting, joining the advantages of both compounds and reducing their limitations in their use as disinfectants. However, it has been poorly studied. Expanding the focus to crude essential oils, the combination of cinnamon, clove, and oregano essential oils with benzalkonium chloride and benzenthonium chloride and cetylpyridinium chloride showed additive bacteriostatic effect against foodborne bacteria on fresh-cut vegetables, achieving a 90–99% higher inhibition than the most active component acting alone [23,24,25,26,27,28]. Shifting the attention to QAC–EOC mixtures in the narrow sense, Pablos et al. [29] assert the existence of synergy in the BAC–carvacrol combination against *Salmonella enterica*, although they do not specify the criteria used to determine the synergistic effect of the combination. Miladi et al. [30] reported a significant reduction in the minimum inhibitory concentration (MIC) of carvacrol and thymol when combined with BAC. Nevertheless, they do not provide a quantitative evaluation of the synergy observed. Just recently, Ziklo et al. [31] quantified the synergistic effect of maltol–DDAC mixtures against *Pseudomonas aeruginosa* and *Staphylococcus aureus* by using commonly employed metrics to asses the combination of antimicrobial compounds. Thus, the literature on this subject is scarce, and the effect (additive or synergistic) of the combinations and its magnitude remains unclear. Furthermore, the efficacy of different combinations involving QACs and EOCs has yet to be experimentally evaluated.

The present work aims to evaluate the combinative effect of binary mixtures (BAC–CAR, BAC–EUG, DDAC–CAR, and DDAC–EUG) against two common food spoilage bacteria: *Escherichia coli* and *Bacillus cereus* [32]. Synergism was quantitatively evaluated through a multifocus approach based on inhibition and biocidal effects. Different EOC–QAC combinations were tested by using the checkerboard assay, and the results were interpreted employing the fractional inhibitory concentration index (FICI) and the fractional bactericidal concentration index (FBCI). Data from the FBCI were fitted to the Bliss synergy model using the software Combenefit [33,34]. Those promising synergistic combinations were further assessed and validated through time–kill curves. As far as we know, this is the first study to assess the antimicrobial effect of BAC–EUG, DDAC–CAR, and DDAC–EUG mixtures.

## 2. Materials and Methods

### 2.1. Bacterial Strains

*B. cereus* (CECT 495) and *E. coli* (CECT 102) were obtained from Colección Española de Cultivos Tipo (CECT, Universidad de Valencia, Spain). These strains were chosen according to their differences at cell wall level and to be used as surrogates of foodborne pathogenic *B. cereus* and *E. coli* O157:H7 strains [35,36]. Stock cultures were cryopreserved at −80 °C in 25% (*v*/*v*) glycerol-supplemented nutritive broth containing 0.5% (*w*/*v*) meat extract (Scharlau SL, Barcelona, Spain), 10% (*w*/*v*) neopeptone (Bacto ™, BD Biosciences, Franklin Lakes, NJ, USA), and 5% (*w*/*v*) NaCl (Emsure R, Merck KGaA, Darmstadt, Germany).

### 2.2. Antimicrobials

Carvacrol (99% purity; product code W224511), eugenol (≥98% purity; product code W246700), and benzalkonium chloride (≥95% purity; product code W246700) were purchased to Sigma-Aldrich (St. Louis, MO, USA). Didecyldimethylammonium chloride (95% purity, product code AB 172768) was purchased to ABCR GmbH & Co KG (Karlsruhe, Germany). All compounds were tested for its antibacterial activity alone and in binary mixtures. Stock solutions (CAR: 800 mg/L; EUG: 3200 mg/L; BAC: 64 mg/L; DDAC: 8 mg/L) were freshly prepared in tryptic soy broth (TSB; Merck Millipore, St. Louis, MO, USA) the day before the experiments and sterilized by autoclaving. Previous experiments discarded the effect of autoclaving on the antimicrobial activity of each compound. When working with EOC, solvents are usually employed to enhance their dispersibility in aqueous solutions. To avoid the interference of a third substance in the binary combination, no solvents were employed in the present study. In accordance with Lambert et al. [37], we have found that the composition of the culture medium increases the dispersibility of the EOCs in the range studied, resulting in a homogeneous emulsion. The above-mentioned stock solutions were vigorously shaken for 2 min before being serially two-fold diluted in TSB to obtain 11 work solutions of each compound (Table 1). Dilutions were vortexed for 1 min at every step.

### 2.3. Determination of Minimum Inhibitory and Bactericidal Concentrations

The minimum inhibitory concentration (MIC) of each antimicrobial was independently assessed by the broth microdilution method following the Clinical and Laboratory Standards Institute (CLSI) guidelines with some modifications [38]. Briefly, overnight cultures were diluted in TSB to an optical density at 600 nm of 0.120, corresponding to 1 × 10^8^ CFUs/mL. From this, a subsequent 1:10 (*v*/*v*) cell suspension was prepared. Then, 80 µL of TSB was added to the wells of Columns 1 to 11 in a 96-well plate. Wells of Column 12 were filled with 180 µL of TSB. After that, 100 µL of two-fold concentrated work solutions were dispensed in Columns 1 to 11. Finally, 20 µL of cell suspension were added to each well, resulting in a final concentration of 1 × 10^6^ CFUs/mL. Plates were incubated at 180 rpm and 37 °C (*E. coli*) or 30 °C (*B. cereus*) for 24 h. MIC was defined as the lowest concentration of the antimicrobial compound that inhibited bacterial growth. Visual inspection was supported by the spectrophotometric measurement of plates at 0 and 24 h on a Thermo Multiskan Spectrum spectrophotometer (Thermo Scientific, Waltham, MA, USA). Microplates were performed by triplicate, and each experiment was conducted three independent times. For each experiment, a fourth plate was prepared by replacing the 20 µL of cell suspension with TSB to exclude a possible increase of turbidity due to other causes than bacterial growth.

The minimum bactericidal concentration (MBC) was determined after MIC reading as follows: from each well without growth, 100 µL aliquots were taken and serially diluted in NaCl 0.9% (*w*/*v*) before being plated by triplicate onto Trypticase Soy Agar (TSA) [39]. Colonies were counted after 24–48 h of incubation at 30 °C (*B. cereus*) or 37 °C (*E. coli*). The MBC was defined as the lowest concentration of antimicrobials that demonstrates at least a 5-log_10_ reduction of the initial inoculum as recommended by the European Standard 1276:2019 [40]. The initial aliquot was not plated to avoid potential antimicrobial carry-over effects. The detection limit was 10 CFUs/mL. The existence of *B. cereus* spores were assessed following the methodology described by Simões et al. [41]. Spores were found at levels below 1 × 10^−5^% of the population of vegetative *B. cereus* cells.

### 2.4. FICI and FBCI Determination by the Checkerboard Assay

The checkerboard assay was employed to test the antimicrobial effect of each binary QAC–EOC mixture. The range of concentrations tested was chosen based on the MIC and MBC of each compound separately and included from 4× MIC to 1/32× MIC. Experiments were performed in 96-well microplates. Briefly, 90 µL of two-fold serial dilutions were added along the rows for one of the compounds, the other compound being added along the columns. The last row and the last column were reserved for testing the compounds alone and were filled with 180 µL of each dilution. The right-bottom well was reserved for growth control and filled with 180 µL. Then, 20 µL of cell suspension prepared as previously described were added to all wells. After incubation, MIC and MBC were determined as described above. Every QAC–EOC combination was tested on three independent experiments and for each experiment, the checkerboard microplates were performed by triplicate (n = 3). Moreover, a non-inoculated fourth microplate was prepared to exclude a possible increase of turbidity not explained by bacterial growth (negative control). Incubation conditions were established as in the previous section: 180 rpm of agitation, 24 h of incubation at 37 °C and 30 °C for *E. coli* and *B. cereus*, respectively.

FICI and FBCI were calculated according to Equation (1) and (2):

(1) $$\begin{array}{c} \mathrm{FICI}=\frac{{\mathrm{MIC}}_{A}\left(\mathrm{combined}\right)}{{\mathrm{MIC}}_{A}\left(\mathrm{alone}\right)}+\frac{{\mathrm{MIC}}_{B}\left(\mathrm{combined}\right)}{{\mathrm{MIC}}_{B}\left(\mathrm{alone}\right)} \end{array}$$

(2) $$\begin{array}{c} \mathrm{FBCI}=\frac{{\mathrm{MBC}}_{A}\left(\mathrm{combined}\right)}{{\mathrm{MBC}}_{A}\left(\mathrm{alone}\right)}+\frac{{\mathrm{MBC}}_{B}\left(\mathrm{combined}\right)}{{\mathrm{MBC}}_{B}\left(\mathrm{alone}\right)} \end{array}$$

FICI and FBCI scores were defined as synergy (≤0.5), indifference (0.5–4), and antagonism (>4.0) [42].

The results from the checkerboard assay were further analyzed using Combenefit 2.021, a software tool designed to facilitate the visualization, analysis, and quantification of the effects of antimicrobial combinations [43]. The data obtained from the calculation of the MBC using the checkerboard assay were normalized as the survival percentage relative to the initial inoculum [44]. The Bliss independence model was chosen as the most appropriate, since it allows for the evaluation of combinations of drugs acting on different pathways or molecular targets and does not assume a fixed dose ratio [45].

### 2.5. Time–Kill Curves

Synergistic combinations with promising FBCI values ≤ 0.5 were further tested following the time–kill curves assay described in the Clinical Microbiology Procedure Handbook [46]. Briefly, 300 mL Erlenmeyer flasks containing 180 mL of TSB were inoculated with 1 mL from a 21 h second-passage culture, resulting in an initial cell concentration of 10^6^ CFUs/mL, and incubated under agitation (200 rpm) at 30 °C (*B. cereus*) or 37 °C (*E. coli*). At predetermined incubation times (0, 2, 4, 8, 12, 16, and 24 h), samples from each flask were several-fold diluted in 0.9% NaCl, and 0.1 mL aliquots were plated, by triplicate, on TSA. Cell colonies were manually counted after 24–48 h of incubation. Synergism was interpreted as a ≥ 2-log_10_ decrease in CFUs/mL compared with the most active single compound. All time–kill cultures were performed by duplicate (two independent experiments).

### 2.6. Modelling Time–Kill Curves

To analyze the results, we propose for the first time a model simulating different behaviors for growth (logistic growth with and without lag phase [47]) and for the bactericidal effect (Chick–Watson and Hom models [48]). Note that parameters are calculated for each curve where the antimicrobial concentration remains constant. Therefore, the dependence with the concentration of disinfection cannot be estimated [49], and the Chick–Watson dynamics $\tilde{k}C^{n}N$ should be simplified to kN with $k=\tilde{k}C^{n}$. Finally, the nested model including all these behaviors reads the following:

(3) $$\frac{dN}{dt}=\left(\frac{a_{0}}{a_{0}+\left(1-a_{0}\right)\exp\left(-\mu \;t\right)}\right)\frac{\mu }{\ln10}\left(1-10^{N-N_{m}}\right)-\frac{K}{\ln10}\;m\;t^{m-1}$$

where *N* is the log10 number of bacterial counts (CFUs/mL). The parameters to be estimated from each time–kill curve are the following: the growth rate μ∈[0,inf]; maximum number of counts $N_{m}\in \left[0,inf\right]$; the level of lag phase $a_{0}\in \left[0,1\right]$, 1 being without lag phase; the K∈[0,inf] the inactivation rate; and the Hom coefficient m∈[0,inf]. (After initial tests, we note that always m∈[0,1] that was the final implementation in the code, being careful to avoid m=0 when t=0.)

The nested model (Equation (3)) allows for all behaviors described in Table 2, depending on the selection of the parameters. Therefore, to avoid overparametrization and detect just the relevant behaviors, we calculated the parameters that minimize the Akaike Information Index (AIC) for the weighted least squares (WLS) with the following optimization problem [50]:

(4) $$\min_{\mu ,N_{m},a_{0},K,m}AIC=n_{d}\ln\frac{\sum_{j=1}^{n_{d}}WLS}{n_{d}}+2\left(n_{p}+1\right)\;with\;WLS={\left(\frac{N_{model}-N_{data}}{0.5}\right)}^{2}$$

$n_{d}$ and $n_{p}$ being the number of data and parameters to be estimated. The differences between model and data are compared in terms of the log_10_ of microbial counts with standard deviation of the data 0.5 (i.e., assuming log-normal distribution for counts [51]). Note that for knowing the number of estimated parameters ($n_{p}$), those that are not relevant for the behavior should not count, so $n_{p}=5$ given a Logistic with Lag plus Hom dynamics, but, for example, $n_{p}=1$ when showing only Chick–Watson decay. Each of the cases can be seen in Table 2.

The code (implemented in Matlab) and data used in this work to conduct the modeling analysis is available in https://zenodo.org/records/11259961, (accessed on 23 May 2024).

## 3. Results and Discussion

### 3.1. Stand-Alone and Combined Antimicrobial Activity of QACs and EOCs

QACs and EOCs demonstrated antimicrobial activity against *E. coli* and *B. cereus* when acting alone (Table 3). MIC and MBC are in line with previous values found in the literature [21,52,53,54].

QACs showed higher performance than EOCs, with MIC and MBC values 1–2 orders of magnitude lower. Both family compounds have a similar antimicrobial spectrum, being effective against Gram-positive and Gram-negative bacteria and fungi—as well as some enveloped and non-enveloped viruses—in a dose-dependent manner [37,52,55]. Similarly, their capacity to combat spores appears to be limited to the ability to reduce their germination, showing no evidence of sporicidal activity [56,57,58]. Nevertheless, the evaluation of the health risks associated with the ingestion of QACs makes EOCs the only ones allowed to be used in the food matrix.

In this work, DDAC showed the highest antimicrobial activity with MIC and MBC values of 4 mg/L against *E. coli*, while the MIC and MBC against *B. cereus* were 2 mg/L and 4 mg/L, respectively. The antimicrobial action of QACs operates through a two-step mechanism: an initial disruption of the cell membrane, followed by denaturing proteins essential for metabolism and growth [59]. In terms of structure–activity relationships, the antimicrobial performance of QACs is correlated with the length of the alkyl group chain. Both BAC and DDAC are generally mixtures of homologous molecules with alkyl chains of different size, ranging from 8 to 18 (C8–C18) carbon atoms in BAC and 8 and 12 (C8–C12) in the case of DDAC [60]. The longer the carbon chains, the stronger the antimicrobial activity, but with a turndown point where this activity begins to decline [59]. This impairment is a consequence of the reduction in the critical micelle concentration of QACs caused by longer alkyl chains which, in turn, diminishes the concentration of QAC monomers with higher antimicrobial activity than micelles [61]. The particular conformation of DDAC, which comprises a pair of alkyl chains (Table 1), may explain its highest antimicrobial activity compared to BAC, formed by a single alkyl group chain [62,63,64].

Among EOCs, our results show a weaker activity of EUG, with a MIC of 1600 mg/L and a MBC of 3200 mg/L against both bacteria, versus the 400 mg/L (MIC) and 400–800 mg/L (MBC) achieved by CAR. Both CAR and EUG act mainly at the microbial cell membrane level, causing its depolarization and the consequent release of intracellular material such as proteins, enzymes, and ions. However, other mechanisms—such as the generation of intracellular reactive oxygen species, changes in DNA structure, and blocking ATPase activity—have also been identified [52,65,66]. In the case of EUG, its antimicrobial activity is also related to its ability to inhibit the production of several enzymes, including amylases and proteases [67]. The differences in the efficacy between CAR and EUG also underlie their different chemical structures. In EOCs, the structure–activity relationship is associated with the hydrophobicity and the activity of the free hydroxyl group. The high hydrophobicity of CAR increases its affinity for lipid layers and facilitates its insertion in the cell membrane. For its part, EUG has low hydrophobicity and a methoxyl group placed in the ortho position, preventing the hydroxyl group from releasing its proton easily [68].

*B. cereus* was more susceptible than *E. coli* to all compounds tested. This finding agrees with the generally established higher tolerance of Gram-negative bacteria to the action of antimicrobial compounds according to the Spaulding classification [69]. The cell wall of Gram-positive bacteria is relatively permeable and hydrophilic, allowing for the free passage of compounds with low molecular weight, such as phenolic compounds [70].

### 3.2. FICI and FBCI

The FICI serves as a means to define the synergy between antimicrobial agents and depends on the MIC values and growth inhibition measurements. However, the adoption of the FICI as a standard for evaluating combinations between antimicrobial compounds has inherent and fundamental limitations, as it tends to overlook their bactericidal properties. Consequently, this drawback may contribute to overlooking those combinations with synergistic bactericidal effect that does not so clearly show an increase in their bacteriostatic potential. The so-called “FICI paradigm” has questioned the validity of FICI as a stand-alone method to evaluate the combination between antimicrobial compounds, pointing to the use of other approaches based on cidality, such as the FBCI or time–kill curves, as more appropriate for considering the bactericidal effect [71].

**Figure 1:**
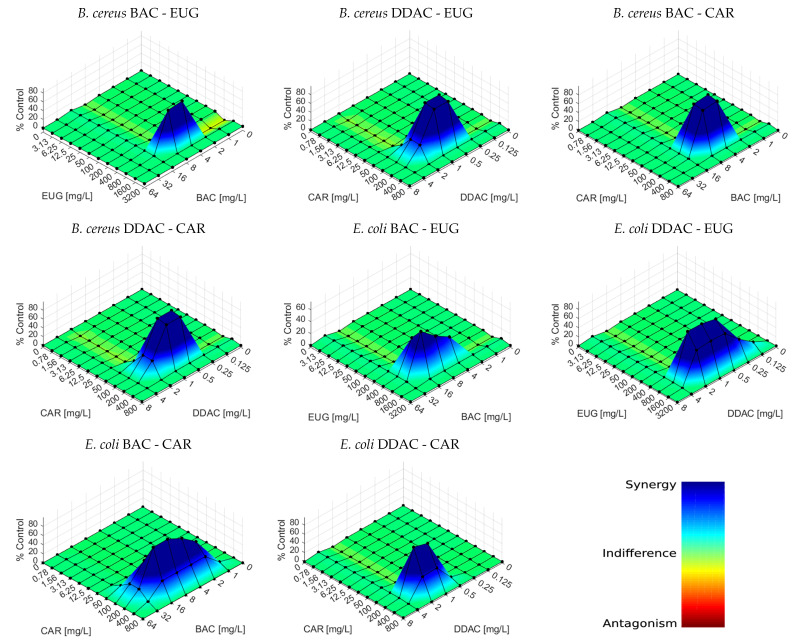
Response surface data analysis of the bactericidal effect of QAC/EOC combinations obtained using Bliss Independence model. Each graph was generated using the mean of three separate experiments, each consisting of three replicates.

The Bliss independence model output supports the empirical data extracted from the calculation of the MBC by demonstrating the existence of a clear synergism between the EOCs and QACs tested (Figure 1). Nevertheless, it appears that a two-fold increase in the antimicrobial concentration—the standard in susceptibility testing—results in a little stepped response. This explains the abruptness of the synergic zones, which is particularly noteworthy in the case of *B. cereus*. This abruptness is also noticeable in the response-surface graph generated by the Bliss model when studying the combination of cinnamaldehyde with either silver nanoparticles [72] or potassium sorbate [73], even despite that the size of the matrix of concentrations tested (6 × 8) is lower than that tested here. Employing a smaller stepped increase would potentially result in smoother curves. The absence of bacteriostatic synergy, as interpreted by the FICI values, may also be attributed to this reason and leads to reconsidering the use of a more stepped increment when evaluating the combined effect of two antimicrobial compounds to increase the resolution of the outcome.

### 3.3. Time–Kill Curves

The FBCI and the FICI provide high-throughput and rapid methods for screening the antimicrobial effect of the interaction of two antimicrobial compounds. However, the interpretation of their results is controversial and depends on the source used. Furthermore, they disregard inactivation dynamics and other phenomena, such as bacterial regrowth.

**Figure 2:**
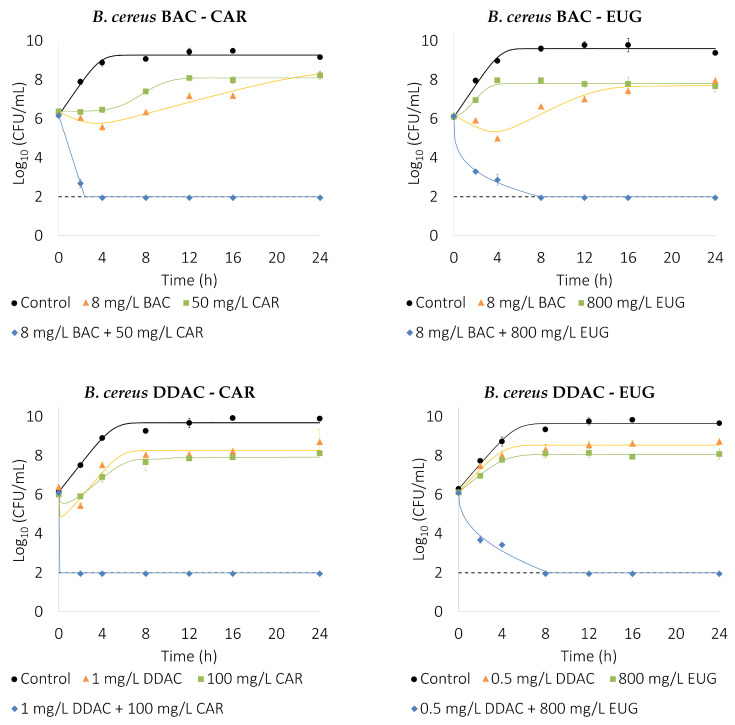
Time–kill curves show the inactivation dynamics of the selected QAC–EOC combinations and their components over *B. cereus*. Model output is depicted by lines, while experimental data is represented by markers. The dashed line represents the detection limit (2-log_10_ CFUs/mL). Error bars depict the standard deviation of two independent experiments with replicates.

**Figure 3:**
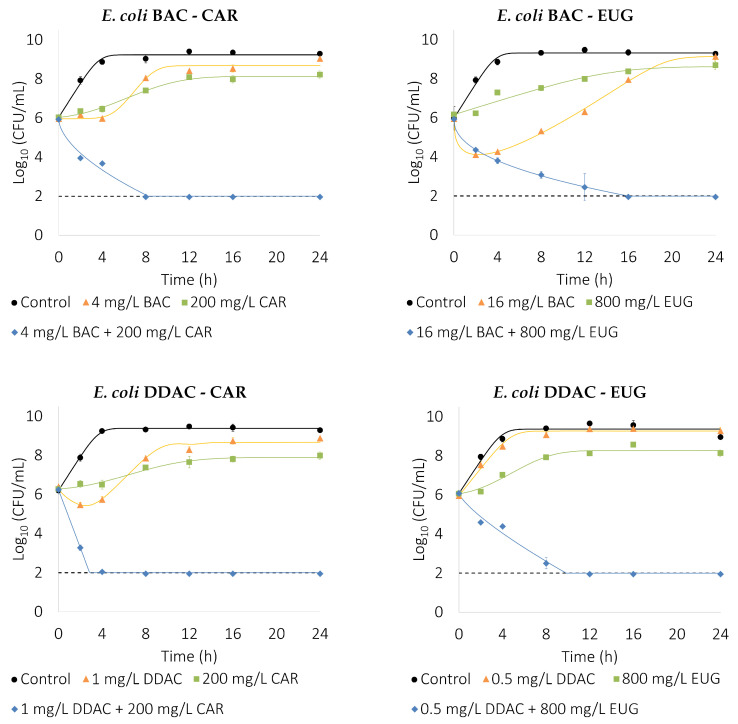
Time–kill curves show the inactivation dynamics of the selected QAC–EOC combinations and their components over *E. coli*. Model output is depicted by lines, while experimental data is represented by markers. The dashed line represents the detection limit (2-log_10_ CFUs/mL). Error bars depict the standard deviation of two independent experiments with replicates.

The experimental data from the time–kill curves (Figure 2 and Figure 3) support the FBCI results and provides valuable information about the time dependence of the inactivation. Even at sub-inhibitory concentrations, when acting alone, the highest QACs concentrations tested (16 mg/L BAC and 1 mg/L DDAC) showed an initial decline in the bacterial population followed by rapid recovery. This was not observed in the case of EOCs, where just lag phase and cell density seems to be affected. All QAC–EOC combinations achieved a decrease of the initial bacterial load under the detection limit of 2-log_10_ CFUs/mL before 16 h. The DDAC–CAR combination seems to be the most efficient, resulting in >4-log_10_ reduction of bacterial population in less than 2 h in the case of *B. cereus* and 4 h for *E. coli*. Interestingly, a biphasic decrease in cell population between 2 and 4 h of exposure is observable for the combinations BAC–EUG and DDAC–EUG in *B. cereus* and BAC–CAR and DDAC–EUG in *E. coli*. This could be attributable to heteroresistance within the bacterial population, suggesting that a fraction of the population might be more recalcitrant to the action of the biocide combination. Similar results were previously found by Park et al. [26] in the inactivation curves of *E. coli* O157:H7 and *Salmonella* Typhimurium exposed to a combination of cetylpyridinium chloride and cinnamon essential oil. The slightly greater reduction in achieved by BAC alone than in the BAC–EUG combination can also be in part explained by this fact. Several plant compounds, including EOCs, can act as resistance-modifying agents [41]. Therefore, EUG could increase the susceptibility to BAC of this more BAC-recalcitrant subpopulation.

The limited number of publications evaluating the combined activity of disinfectants using time–kill curves makes it challenging to compare them with the combinations tested here in terms of the time needed to achieve a given level of inactivation. Waltimo et al. [74] studied the combined activity of the disinfectants sodium hypochlorite, calcium hydroxide, chlorhexidine digluconate, and iodine potassium iodide on *Candida albicans* employing time–kill curves. With the exception of the chlorhexidine-calcium hydroxide combination, which achieved a 50% reduction in the initial cell density, and the iodine-calcium hydroxide combination, which achieved a 5% reduction, all other combinations demonstrated a 100% reduction in cell density after just one hour of exposure. Nevertheless, this reduction was comparable to that achieved by the individual components acting alone, so it is not possible to define the combinations as synergistic. In Noel et al.’s study [75], the antimicrobial activity of BAC-chlorocresol and polyhexamethylene biguanide-chlorocresol combinations demonstrated a clear synergistic action against *Enterococcus faecalis* and *Staphylococcus aureus*, achieving a logarithmic reduction of the CFUs/mL between 2 and 4 times greater than that achieved by the most active component acting alone. The time required to achieve such a degree of inactivation was between approximately 6 h.

The analysis by fitting each time–kill curve to the dynamic model (Equation (3)), using the Akaike Information Index as an objective function, selects the minimum behavior to represent the data. Table 4 shows the different behavior for all of the curves as well as the estimated parameters. Note that, whereas control experiments always show only growth with logistic dynamics, antimicrobials have many different effects. Under the presence of a single antimicrobial, the following effects are observed: (1) a bacteriostatic effect (only logistic growth) in, for example, *B. cereus* under 0.5 mg/L DDAC and 800 mg/L EUG, with a decrease in the growth velocity and maximum number of cells with respect to the control, (2) a bacteriostatic effect inducing an initial delay in growth (logistic growth with lag phase) in, for example, *B. cereus* under 100 mg/L CAR, where there is a long initial lag phase before starting bacterial growth, and (3) a mixture of bacteriostatic and bactericidal effects, where growth can be with or without a lag phase and bactericidal effect, and this may be better represented with Chick–Model or Hom dynamics. In combinations, only a bactericidal effect is observed, either with a linear decay in the logarithmic scale, and therefore represented with the simple Chick–Watson model with n=1 (for example for 4 mg/L BAC + 100 mg/L CAR for *B. cereus*, or a slower than linear decay, represented by the Hom model (for example 8 mg/L BAC + 800 mg/L EUG for *B. cereus*).

The underlying mechanisms behind the enhanced antimicrobial performance of the combinations among surfactants and essential oils or their constituents have not yet been completely elucidated, but theoretical postulations have been proposed. At the macroscopic level, the emulsification increases the wettability of EOCs and facilitates the coverage of food surfaces while at the microscopic scale, the cationic charges supplied by QACs in QAC–EOC combinations facilitate their penetration into the negatively charged bacterial cell membrane [27,76].

The antimicrobial activity of the combinations is largely dependent on the type of surfactant and the essential oil or EOC. Thus, the use of anionic surfactants such as polysorbate 80 results in a lower antimicrobial activity than that demonstrated by the essential oil when acting alone [27,28]. El-Sayed et al. [77] suggested that the reason for this antagonistic effect lies in the formation of hydrogen bonds between the hydroxyl groups of polysorbate 80 and the phenolic compounds responsible for the antimicrobial activity of certain essential oils.

Controversially, the combination of maltol and polysorbate 80 shows no antagonism; rather, it shows a synergistic antimicrobial effect [31]. However, it should be noted that maltol is a pyrone and its structure differs significantly from that of a phenolic compound. Interestingly, maltol is aqueous soluble, so the synergistic effects reported when combined with polysorbate 80 or DDAC are not attributed to increased EOC dispersibility. Instead, they argued that the effect likely results from maltol’s chelating activity on Mg^2+^ and Ca^2+^ cations, which are crucial for the integrity and stability of the bacterial outer membrane. This chelating activity potentially facilitates the disruption of the cell envelope and the access of maltol and polysorbate/DDAC to their intracellular targets. Apparently, the quenching of EOC antimicrobial activity does not occur when employing cationic surfactants, reinforcing the present results and the validity of QACs as a better option to increase both the dispersibility and antimicrobial action in EOC–surfactant combinations.

Based on the mode of action and the physical properties of the compounds employed in the present study and on the previous knowledge, we may venture that the synergistic effects found among BAC–CAR, BAC–EUG, DDAC–CAR, and DDAC–EUG may lie in the same mechanisms, i.e., (1) an increase in the dispersibility of EOCs due to the surfactant properties of QACs, facilitating their interaction with the cell surface, and (2) the formation of mixed QAC–EOC micelles or dimers, which may facilitate entry into the cell interior and, thereby, attack intracellular targets without requiring as high a concentration to achieve this, as would be necessary when acting alone. It seems unlikely that chelating activity over Mg^2+^ and Ca^2+^ cations of a bacterial envelope will occur in the case of CAR–QAC combinations [78]. However, we could not discard this possibility for EUG–QAC combinations. Nevertheless, further research is required to specifically elucidate the underlying mechanisms of the synergy among the compounds of the present study.

While the direct application of QACs in food matrices is not feasible due to safety concerns, the potential for the use of QAC–EOC combinations in the sanitization and disinfection of utensils, surfaces, and facilities within the food industry is significant. The enhanced bactericidal efficacy observed in our study suggests that these combinations could lead to more effective and safer disinfection protocols by reducing the concentration of QACs while increasing the antimicrobial activity. In practical terms, the incorporation of these findings into real-world practices could involve the development of specialized disinfectant formulations tailored for use in food processing environments. In addition, our study highlights the critical importance of assessing bactericidal effects alongside bacteriostatic effects when evaluating antimicrobial synergy. This comprehensive approach ensures that the selected combinations not only inhibit bacterial growth but also effectively eliminate bacterial populations.

## 4. Conclusions

The present study demonstrates bactericidal synergy in two mixtures of quaternary ammonium compounds and essential oil constituents. Synergistic interaction was supported by the standards procedures fractional bactericidal concentration index and time–kill curves. The fractional inhibitory concentration index did not show any evidence of synergy, emphasizing the importance of considering both the bacteriostatic and bactericidal effects when evaluating the performance of biocide combinations. The most significant efficacy was shown by carvacrol–didecyldimethylammonium chloride and eugenol–didecyldimethylammonium chloride combinations, leading to a 5-log_10_ reduction in 2 h (*B. cereus*) and 4 h (*E. coli*) of the initial bacterial population. In contrast, eugenol–benzalkonium chloride exhibited the lowest bactericidal efficiency, requiring up to 16 h to achieve an equivalent level of reduction. Quaternary ammonium compounds, unlike essential oil constituent, are not authorized for direct use in food. Their application is permitted in the formulation of disinfectants specifically intended to be employed in food industry facilities. Nevertheless, the combination of quaternary ammonium compounds and essential oil constituents overcome the individual disadvantages associated with each compound, and emerges as a promising and effective approach for the management of bacterial growth. Future work will be aimed at clarifying the mode of action of these combinations on bacterial physiology and structure, as well as in situ studies in actual food processing environments to demonstrate the applicability of these combinations in the food industry.

## Figures and Tables

**Table 1:** Chemical structure and tested concentrations of the studied compounds.

	Compound	Chemical Structure	Tested Concentrations (mg/L)
QACs	BAC	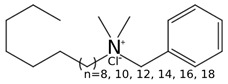	1, 2, 4, 8, 16, 32, 64
	DDAC	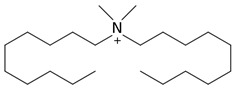	0.13, 0.25, 0.50, 1, 2, 4, 8
EOCs	Carvacrol	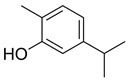	0.78, 1.56, 3.13, 6.25, 12.50, 25, 50, 100, 200, 400, 800
	Eugenol	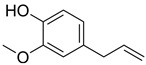	3.13, 6.25, 12.50, 25, 50, 100, 200, 400, 800, 1600, 3200

**Table 2:** Outline of the different models and behaviors when changing parameter values in Equation (3). X: parameter to be estimated; -: not applicable (any value can be introduced as it does not affect the dynamics).

Effect	Behavior	*µ*	*N_m_*	*a*_0_	*K*	*m*	*n_p_*
Only Growth	Logistic	X	X	1	0	-	2
	Logistic + Lag	X	X	X	0	-	3
Only Decay	Chick–Watson	0	-	-	X	-	1
	Hom	0	-	-	X	X	2
Growth & Decay	Logistic + Chick–Watson	X	X	1	X	-	3
	Logistic + Hom	X	X	1	X	X	4
	Logistic + Lag + Chick–Watson	X	X	X	X	-	4
	Logistic + Lag + Hom	X	X	X	X	X	5

**Table 3:** MIC and MBC of the tested compounds against *E. coli* and *B. cereus*, acting alone or combined, together with the FICI and the FBCI, and the log_10_ reduction achieved for each combination. ^I^: Indifference (>0.5); ^S^: Synergism (≤0.5) [42].

	Combination		MIC (mg/L)		MBC (mg/L)		FICI	FBCI	Log_10_ Reduction
			Alone	Combined	Alone	Combined			
*B. cereus*	BAC + EUG	BAC	32	8	32	8	0.75 ^I^	0.50 ^S^	5.06 ± 0.43
		EUG	1600	800	3200	800			
	DDAC + EUG	DDAC	2	0.5	2	0.5	0.75 ^I^	0.50 ^S^	5.77 ± 0.20
		EUG	1600	800	3200	800			
	BAC + CAR	BAC	32	16	32	8	0.63 ^I^	0.38 ^S^	5.75 ± 0.08
		CAR	400	50	400	50			
	DDAC + CAR	DDAC	2	1	4	1	0.75 ^I^	0.50 ^S^	5.68 ± 0.23
		CAR	400	100	400	100			
*E. coli*	BAC + EUG	BAC	32	4	64	16	0.63 ^I^	0.50 ^S^	5.38 ± 0.37
		EUG	1600	800	3200	800			
	DAC + EUG	DDAC	4	0.5	4	0.5	0.63 ^I^	0.38 ^S^	5.44 ± 0.35
		EUG	1600	800	3200	800			
	BAC + CAR	BAC	32	2	64	4	0.56 ^I^	0.31 ^S^	5.57 ± 0.25
		CAR	400	200	800	200			
	DDAC + CAR	DDAC	4	1	4	1	0.75 ^I^	0.50 ^S^	5.69 ± 0.22
		CAR	400	200	800	200			

**Table 4:** Estimated behavior in the dynamic mathematical model describing the time–kill curves.

			Parameters				
	Experiment	Model	μ	$N_{m}$	$a_{0}$	K	m
	(Compound [mg/L])		$h^{-1}$	${\mathrm{Log}}_{10}(\frac{\mathrm{CFUs}}{\mathrm{mL}})$	−	$h^{-m}$	−
*B. cereus*	Control	Logistic	1.90	9.60	1	0	-
	BAC [8]	Logistic with Lag + Chick–Watson	1.39	8.02	0.01	0.73	1
	EUG [800]	Logistic with Lag	1.90	7.82	0.19	0	-
	BAC [8] + EUG [800]	Hom	0	-	-	5.38	0.28
	Control	Logistic	1.87	9.27	1	0	-
	BAC [8]	Logistic with Lag + Chick–Watson	1.04	9.03	0.05	0.70	1
	CAR [50]	Logistic with Lag	1.11	8.10	0.00	0	-
	BAC [8] + CAR [50]	Chick–Watson	0	-	-	4.02	1
	Control	Logistic	1.64	9.69	1	0	-
	DDAC [1]	Logistic + Hom	1.64	8.28	1	214.53	0.00
	CAR [100]	Logistic + Hom	1.64	7.97	1	2.51	0.42
	DDAC [1] + CAR [100]	Chick–Watson	0	-	-	471.91	1
	Control	Logistic	1.50	9.65	1	0	-
	DDAC [0.5]	Logistic	1.37	8.54	1	0	-
	EUG [800]	Logistic	1.11	8.06	1	0	-
	DDAC [0.5] + EUG [800]	Hom	0	-	-	3.79	0.43
*E. coli*	Control	Logistic	2.03	9.24	1	0	-
	BAC [4]	Logistic with Lag	1.70	8.68	0.00	0	-
	CAR [200]	Logistic with Lag	0.62	8.14	0.21	0	-
	BAC [4] + CAR [200]	Hom	0	-	-	2.66	0.58
	Control	Logistic	2.02	9.38	1	0	-
	DDAC [1]	Logistic with Lag + Hom	1.74	8.74	0.01	1.59	0.59
	CAR [200]	Logistic with Lag	0.51	7.90	0.21	0	-
	DDAC [1] + CAR [200]	Chick–Watson	0	-	-	3.42	1
	Control	Logistic	2.05	9.32	1	0	-
	BAC [16]	Logistic + Hom	1.23	9.27	1	5.12	0.39
	EUG [800]	Logistic	0.40	8.63	1	0	-
	BAC [16] + EUG [800]	Hom	0	-	-	2.64	0.44
	Control	Logistic	1.95	9.36	1	0	-
	DDAC [0.5]	Logistic	1.62	9.27	1	0	-
	EUG [800]	Logistic with Lag	0.81	8.27	0.23	0	-
	DDAC [0.5] + EUG [800]	Hom	0	-	-	1.62	0.77

## Data Availability

The original contributions presented in the study are included in the article. Further inquiries can be directed to the corresponding author. Data and code (Matlab) for simulations are available in a publicly accessible repository https://zenodo.org/records/11259961 (accessed on 23 May 2024).
